# A stepwise strategy integrating metabolomics and pseudotargeted spectrum–effect relationship to elucidate the potential hepatotoxic components in *Polygonum multiflorum*

**DOI:** 10.3389/fphar.2022.935336

**Published:** 2022-08-26

**Authors:** Yunfei Song, Jianbo Yang, Xiaowen Hu, Huiyu Gao, Pengfei Wang, Xueting Wang, Yue Liu, Xianlong Cheng, Feng Wei, Shuangcheng Ma

**Affiliations:** ^1^ School of Chinese Materia Medica, Beijing University of Chinese Medicine, Beijing, China; ^2^ Institute for Control of Chinese Traditional Medicine and Ethnic Medicine, National Institutes for Food and Drug Control, Beijing, China

**Keywords:** polygonum multiflorum, hepatotoxicity, pseudotargeted spectrum–effect relationship, plant metabolomics, mathematical model

## Abstract

*Polygonum* *multiflorum* (PM) Thunb., a typical Chinese herbal medicine with different therapeutic effect in raw and processed forms, has been used worldwide for thousands of years. However, hepatotoxicity caused by PM has raised considerable concern in recent decades. The exploration of toxic components in PM has been a great challenge for a long time. In this study, we developed a stepwise strategy integrating metabolomics and pseudotargeted spectrum–effect relationship to illuminate the potential hepatotoxic components in PM. First, 112 components were tentatively identified using ultraperformance liquid chromatography-quadrupole-time-of-flight-mass spectrometry (UPLC-Q-TOF-MS). Second, based on the theory of toxicity attenuation after processing, we combined the UPLC-Q-TOF-MS method and plant metabolomics to screen out the reduced differential components in PM between raw and processed PM. Third, the proposed pseudotargeted MS of 16 differential components was established and applied to 50 batches of PM for quantitative analysis. Fourth, the hepatocytotoxicity of 50 batches of PM was investigated on two hepatocytes, LO2 and HepG2. Last, three mathematical models, gray relational analysis, orthogonal partial least squares analysis, and back propagation artificial neural network, were established to further identify the key variables affecting hepatotoxicity in PM by combining quantitative spectral information with toxicity to hepatocytes of 50 batches of PM. The results suggested that 16 components may have different degrees of hepatotoxicity, which may lead to hepatotoxicity through synergistic effects. Three components (emodin dianthrones, emodin-8-*O-β*-D-glucopyranoside, PM 14-17) were screened to have significant hepatotoxicity and could be used as toxicity markers in PM as well as for further studies on the mechanism of toxicity. Above all, the study established an effective strategy to explore the hepatotoxic material basis in PM but also provides reference information for in-depth investigations on the hepatotoxicity of PM.

## 1 Introduction

*Polygonum multiflorum* (PM) Thunb., known as one of the “Four Great Herbs” in ancient China (PM, Ginseng, Ganoderma lucidum, Cordyceps sinensis), is widely used in many Chinese prescriptions and patent medicines due to its remarkable therapeutic effects. As early as the Song dynasty, the historical Chinese medicine document “Kai Bao Ben Cao” recorded the pharmacological efficacy of PM as “strengthen muscles and bones, benefit the essence, prolong life” (Lei et al., 2015; Teka et al., 2021). With different therapeutic effects, in general, PM can be divided into raw and processed PM in clinical applications. The Chinese pharmacopoeia states that raw PM has the effects of detoxification, eliminating carbuncles, moistening the intestine, and relieving constipation, while the processed product has been used mainly to tonify the liver and kidney, nourish blood, blacken hair, strengthen the body, dissolve turbidity, and lower blood lipid levels (Chinese Pharmacopoeia Commission, 2020). Meanwhile, modern pharmacological research has shown that the main active ingredients of PM are stilbene glycosides, anthraquinones, glycosides, phospholipids, flavonoids and others, which significantly contribute to delaying senescence, preventing cardiovascular diseases, tonifying the kidney and hair, improving intelligence, enhancing immune function, protecting the liver, moistening the intestine, and defecating as well as have antibacterial and antiinflammatory effects (Lin et al., 2015; Liu et al., 2018).

However, since the 1990s, there has been a rapid increase in reports of liver damage caused by PM, which has attracted attention at home and abroad (But et al., 1996; Park et al., 2001; Han et al., 2019). Thereafter, the drug supervision and administration departments of the United Kingdom, Japan, and China successively issued warnings or regulatory measures for the risk of liver damage from PM and its preparations. In fact, the ancient textbook “Ben Cao Hui Yan” recorded “*Polygonum multiflorum*, taste bitter, astringent, flavor mild, slightly toxic.” Processed PM can significantly relieve the toxicity and change the efficacy of PM, and a relatively complete processing method for PM was used in the Song dynasty. Modern pharmacological studies have also confirmed that processing can greatly reduce the risk of hepatotoxicity of PM. However, the chemical composition of PM is complex and diverse, and PM mainly includes stilbenes, anthraquinones, anthranone, glycosides, phospholipids, flavonoids, and tannins (Lin et al., 2015; Teka et al., 2021). The issue of which components of PM cause hepatotoxicity remains a major subject that needs to be addressed.

In general, the traditional research approach was to first isolate and identify compounds from PM and then to evaluate the compounds for hepatotoxicity *in vivo* or *in vitro*. This process was time-consuming and laborious but also neglected the synergistic toxic effects of the compounds in PM, so the hepatotoxicity of PM could not be comprehensively evaluated. Therefore, it was imperative to develop an effective scientific strategy to efficiently screen out the toxic components of PM.

In recent years, with the development of high-resolution mass spectrometry (MS) and metabolomics techniques, ultraperformance liquid chromatography-quadrupole-time-of-flight-MS (UPLC-Q-TOF-MS) has made it possible to characterize complex components in PM in a short time, and metabolomics combined with chemometrics has enabled the rapid search for differential markers between raw and processed PM (Liu et al., 2016; Shang et al., 2021). Moreover, spectrum–efficiency relationship research has opened a new window for the evaluation of modern traditional Chinese medicine (TCM), which combines the complex chemical information of TCM with pharmacological efficacy and screens the important features related to the efficacy by means of chemometric statistical methods or machine learning (Zhang et al., 2018; Rao et al., 2022). In particular, great progress has been made in the joint analysis of the spectrum–effect relationship based on fingerprinting and pharmacodynamics for illuminating active ingredient markers in complex TCMs. However, the lack of ultraviolet absorption of many compounds and trace components and the lack of standard reference materials pose serious challenges for absolute quantification. Xu’s proposed pseudotargeted metabolomics, establishing a scheduled MRM method for the semiquantification of metabolites, gave us an inspiration of what to do (Luo et al., 2015; Zheng et al., 2020). Compared with previous methods, the established UPLC-coupled scheduled MRM method was a more powerful technique with significant advantages of high sensitivity, wide universality, low matrix effects, and accurate quantification.

**FIGURE 1:**
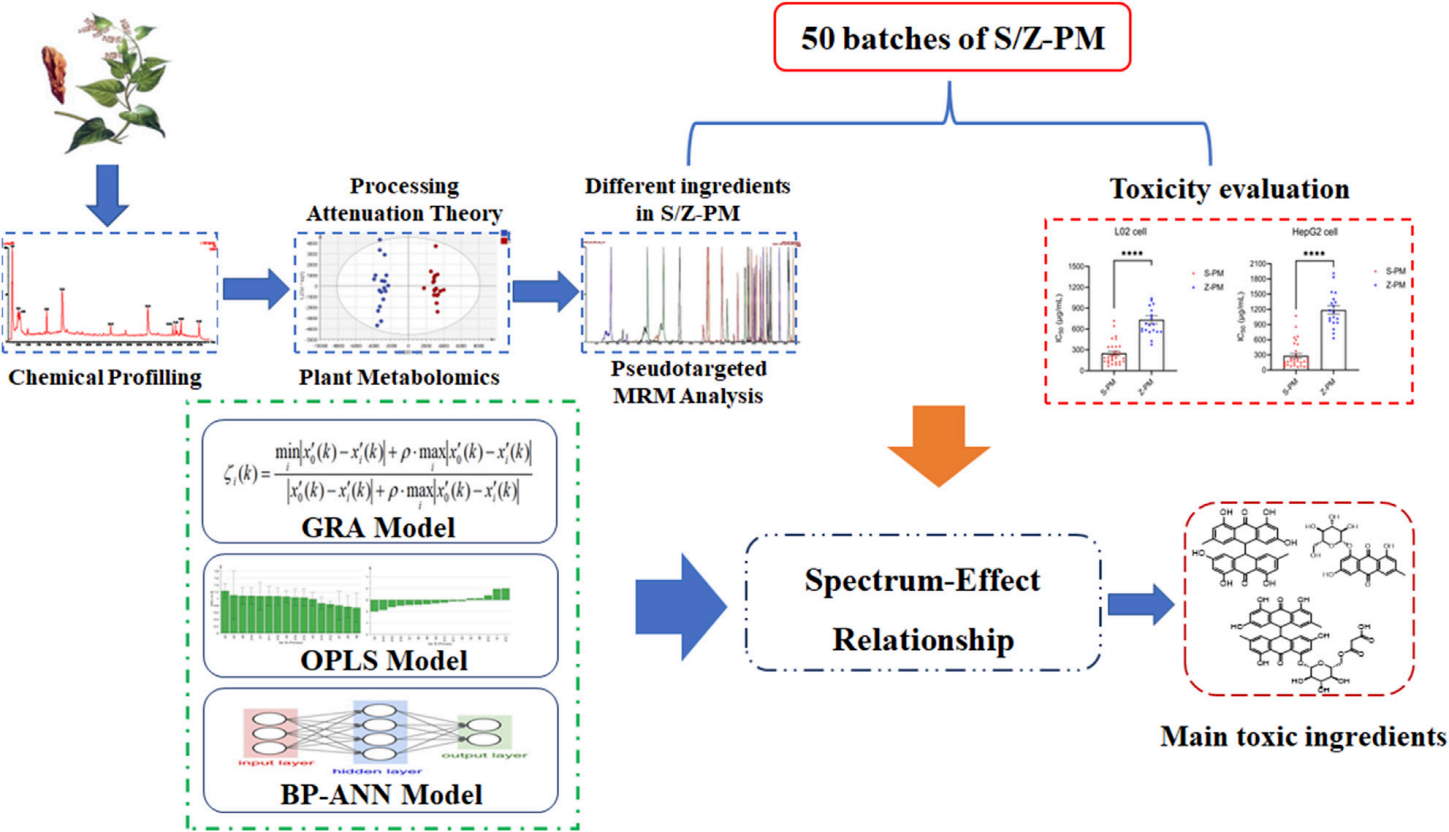
Strategy of integrating metabolomics and pseudotargeted spectrum–effect relationship in this study.

In the current study, a stepwise strategy integrating metabolomics and pseudotargeted spectrum–effect relationship was set up to clarify the potential hepatotoxic components in PM (Figure 1). First, the chemical composition of PM was comprehensively characterized using UPLC-Q-TOF-MS. Second, based on the theory of detoxification after PM processing, the distinctive differential components between raw and processed PM were screened out using plant metabolomics. Third, the proposed pseudotargeted MRM semiquantitative profiles of the differential marked components were established in different batches of PM. Fourth, the toxicity of various batches of PM to the hepatocytes L02 and HepG2 was investigated. At last, gray relational analysis (GRA), orthogonal partial least squares (OPLS) analysis, and back propagation artificial neural network (BP-ANN) models were established to correlate the peak areas of pseudotargeted spectra with the IC_50_ values of toxicity to further obtain the hepatotoxic components in PM.

## 2 Materials and methods

### 2.1 Materials and reagents

Methanol and acetonitrile of LC/MS grade were obtained from Merck (Darmstadt, Germany). High-performance liquid chromatography–grade ethanol and dimethyl sulfoxide (DMSO) were obtained from Sinopharm Chemical Reagent Co., Ltd (Shanghai, China). Ultra-pure water was prepared using a Milli-Q system (Billerica, MA, United States). Standard products of stilbene glycoside, emodin, etc., were provided by the China National Institute for Food and Drug Control. Physcion-8-*O-β*-D-glucopyranoside, physcion-1-*O-β*-D-glucopyranoside, and aloe-emodin-3-hydroxymethyl-*β*-D-glucopyranoside were purchased from Standard Technology Co., Ltd (Shanghai, China). The purity of all standards was above 98%. Formic acid was acquired from Tokyo Chemical Industry Co., Ltd. (Tokyo, Japan). A 0.22-µm filter membrane was purchased from Dikema Technology Co., Ltd. (Beijing, China).

The hepatic cell lines HepG2 and L02 were obtained from the cell bank of the Chinese Academy of Sciences (Shanghai, China). Dulbecco’s Modified Eagle Medium (DMEM) and Roswell Park Memorial Institute (RPMI) 1640 culture medium (Biological Industries, Israel), fetal bovine serum (FBS; Biosera, France), 1% penicillin-streptomycin (Targetmol, China), and 0.25% trypsin-ethylenediaminetetraacetic acid (Wisent, Canada) were used for cell culture. Staurosporine (STSP) and CCK-8 reagent were obtained from Targetmol (Shanghai, China). A total of 384 cell culture plates were purchased from Jet Bio-Filtration Co., Ltd. (Guangzhou, China). The Victor Nivo multimode plate reader was from PerkinElmer (Shanghai, China).

Sample A: 36 batches of raw and processed PM from different origins or batches. In total, 0.1 g was taken from each batch to make 10 portions of mixed samples as quality control (QC). Sample B: 30 batches of raw PM and 20 batches of processed PM. Samples A and B all met the requirements of the Chinese pharmacopoeia. The samples were stored at the China National Institute for Food and Drug Control (Beijing, China). Detailed sample information can be found in Supplementary Tables S1, S2.

### 2.2 Sample and standard solution preparation

Sample A (46 samples in total, filtered through a No. 3 sieve): The sample (1.0 g) was weighed precisely and placed in a 50-mL conical flask. Then, 50 mL of 70% ethanol was added, and the mixture was weighed, sonicated for 30 min, cooled, and replenished. The extracted solution was collected for UPLC-Q-TOF-MS analysis.

The standard solution was prepared by weighing 1 mg of standard powder and adding 2 ml of methanol solution to dissolve it for the qualitative test. All standard and sample solutions were filtered through 0.22-μm Millipore filtration before injection.

Sample B (50 samples): 20 g of PM was weighed and extracted with 300 ml of 70% ethanol three times for 30 min each time. Then, the extracted solutions were combined and concentrated under pressure and subsequently freeze-dried to powder. The dry extract powder weighing 40 mg was dissolved in 40 ml of 70% ethanol solution for UPLC-qqq-MS/MS analysis. Of note, 30 mg of dried extract powder was weighed precisely and prepared as a storage solution of 200 mg/ml. Then, a series of concentrations of working solutions (1,000, 400, 160, 64, 25, 10, and 4 μg/ml) was obtained by gradient dilution with culture medium for the *in vitro* cytotoxicity assay.

### 2.3 Ultraperformance liquid chromatography-quadrupole-time-of-flight-mass spectrometry analysis

#### 2.3.1 Chemical composition characterization

The extract solution of the PM mixed sample in sample A was analyzed using UPLC-Q-TOF-MS. Analysis was performed using an Acquity™ UPLC Class I system equipped with a photodiode array (PDA) detector and Q-TOF SYNAPTG2-Si (Waters, Manchester, United States). Chromatographic conditions: The temperature of the column and autosampler was maintained at 40°C and 6°C. The flow rate was 0.3 ml/min, and the injection volume was 1 µl. The binary mobile phase contained solvent A (0.1% FA in deionized water, v/v) and solvent B (methanol, LC-MS grade). The peptides of the elution gradient were initial 10% B, linear gradient 40% B (22 min), 70% B (33 min), 100% B (44–46 min), 10% B (46.2 min), and holding 10% B to 50 min. The PDA detector used 3D range from 190 to 400 nm. MS conditions: The UPLC-MS system was operated in the negative ion and MS^E^ data acquisition mode. Experimental parameters were set as follows: capillary voltage at −2.5 kV (ESI^−^); source temperature at 115°C; cone voltage at 40 V; ramp trap MS collision energy of 20–50 V; desolvation temperature at 450°C; cone gas flow of 50 L/h; desolvation gas flow of 900 L/h; and scan range of *m/z* 50–1,500 Da. At the same time, an external reference consisting of 1.0 ng/ml solution of leucine enkephalin was used to produce a reference ion at *m/z* 554.2615 Da ([M-H]^−^) in negative ion mode for real-time mass correction during acquisition. The obtained mass spectrometric data were analyzed using UNIFI software in combination with a self-built database of PM compounds and reference standards as well as fragment ion matching strategies to fully characterize the components of PM.

#### 2.3.2 Plant metabolomics analysis

Processed sample A (*n* = 46) was analyzed using UPLC-Q-TOF-MS under the same chromatographic and mass spectrometric conditions as in Section 2.3.1. The acquired data were further deconvolved into a data matrix (Rt-*m/z*-intensity) by Progenesis QI software (Waters, Milford, MA, United States). After further data preprocessing, chemometric (principal component analysis (PCA), PLS-DA, OPLS-DA) analysis was performed using Simca-P 14.1 software. Combining univariate statistical analysis of *P* and FC values with multivariate statistical analysis of VIP values further screened out the differential ions between raw and processed PM.

### 2.4 Ultraperformance liquid chromatography-qqq-MS/MS analysis

#### 2.4.1 Scheduled MRM method development

The scheduled MRM ion pairs were established based on the differential ions and secondary fragment ions of PM from the results of Section 2.3.2. Then, combined with the composition identification results of PM, the MRM ion pairs were further confirmed, and the proposed pseudotarget MRM method was constructed. This method was used to perform semiquantitative analysis in sample B, and the peak area data of the marker components were acquired.

The analysis of samples was performed using a Waters Acquity™ UPLC I-Class system equipped with a Xevo TQ-XS mass spectrometer (Waters, Milford, MA, United States). The chromatographic column and chromatographic separation conditions were the same as the conditions of the previous UPLC-Q-TOF-MS method. The optimal MS conditions were as follows: capillary voltage at 2.5 kV under negative mode; source temperature at 150°C; desolvation gas temperature at 500°C; desolvation gas flow at 850 L/h; and cone gas flow at 150 L/h. Ion pairs and CV and CE parameters are detailed in Table 1.

**TABLE 1:** Optimized ion pairs and CV and CE parameters of 16 compounds.

No.	Compounds	Ion pair (*m/z*)	CV	CE
X1	Catechin	289.07 > 203.07	30	29
X2	Epicatechin	289.07 > 203.07	30	29
X3	Torachrysone-8-*O-β*-D-glucopyranoside	407.13 > 245.08	30	33
X4	7-acetyl-3,8-dihydroxy-6-methyl-1-naphthyl-*β*-D-glucopyranoside	393.12 > 231.06	30	33
X5	Epicatechin-3-*O*-gallate	441.08 > 289.07	30	34
X6	Emodin-8-*O-β*-D-glucopyranoside	431.1 > 269.04	30	34
X7	Emodin bianthrones	509.12 > 253.75	30	31
X8	Emodin-physcion bianthrones	523.14 > 253.83	30	30
X9	Physcion bianthrones	537.15 > 254.73	30	41
X10	2,3,5,4′-tetrahydroxystilbene-2-*O-β*-D-(2-*O*-monogalloylesters)-glucopyranoside	557.13 > 243.06	30	30
X11	polygonibene E	581.16 > 243.06	30	30
X12	Polygonumnolides C1-C4	671.18 > 416.11	30	26
X13	Polygonumnolides A1-A4	685.19 > 416.11	30	26
X14	PM 14-17	757.17 > 458.12	30	31
X15	PM 22-25	933.24 > 458.12	30	33
X16	PM 5	919.23 > 458.12	30	33

The pseudotargeted MRM method was applied for semiquantitative comparison of PM samples (raw PM: S1-S30, processed PM: Z1-Z20).

#### 2.4.2 Method validation

The developed UPLC-MS/MS method was validated with sample Z-1 as an example in terms of specificity, repeatability, precision, linearity, and stability. Specificity was evaluated by comparing samples with the negative control. Repeatability evaluation was carried out by analyzing six replicate samples independently. Precision was investigated by six consecutive injections of the same sample. Linearity was constructed by fitting the peak area of each compound under the injection of 0.5, 1, 1.5, 2, 2.5, and 3 µl of one sample. The same sample was injected at 0, 6, 12, 24, and 30 h to verify the stability. The relative standard deviation (RSD) of the peak area of the characteristic peaks was used to evaluate the results.

### 2.5 Hepatotoxicity assay *in vitro*

Two types of hepatocytes, L02 and HepG2, were used to assess the hepatotoxicity of PM extract *in vitro*. L02 and HepG2 cells were inoculated in 384-well cell plates (density: HepG2 1,000 cells/well; L02 800 cells/well) with 40 µl of cell suspension per well and were incubated overnight at 37°C in a 5% CO_2_ incubator. HepG2 cells were cultured in DMEM containing 10% FBS and 100 U/mL penicillin and streptomycin, while L02 cells were cultured in RPMI 1640 medium. On the day of the experiment, 10 µl of compound working solution (sample B, PM extracting solution of 0.064, 0.32, 1.6, 8, 40, 200, and 1,000 μg/ml) was added to each well according to the experimental requirements, and this was cultivated at 37°C for 72 h with 5% CO_2_ shielded from light. At the end of the incubation, 5 µl of CCK8 reagent was added to the cell plates, and this were incubated for 4 h with 5% CO_2_ at 37°C. The absorbance at 450 nm was measured, and the inhibition rate was calculated according to the following equation:

$$\text{Inhibition ratio }\left(\text{\%}\right)\;=\;\left({\text{OD}}_{\text{S }}-{\text{OD}}_{\text{NC}}\right)/\left({\text{OD}}_{\text{STSP }}-{\text{OD}}_{\text{NC}}\right)\;\times \;100\text{\%}$$

where OD_S_ stands for the absorbance of the working solution (cell + medium + compound to be tested); OD_NC_ stands for the absorbance of the negative control (cell + medium + DMSO); and OD_STSP_ stands for the absorbance of the positive control (cell + medium + 10 μM STSP).

According to the inhibition ratios of the compounds, the IC_50_ values (the concentration corresponding to 50% of the maximum inhibition response) were calculated from the dose–response curves using GraphPad Prism 9.0. The experiment was performed three times in parallel, and finally, the mean IC_50_ value was obtained for each sample.

### 2.6 Spectrum–effect relationship analysis

#### 2.6.1 Gray relational analysis

GRA is a method to determine the degree of association between factors based on the similarity of the geometry of the change curves in each factor. As a simple and effective method, GRA has been widely used in the evaluation of spectrum–effect relationship in TCM (Wang et al., 2018; Ma et al., 2020). In this study, the peak area of each feature was taken as the comparison series, and the 1/IC_50_ value of the cytotoxicity assessment index was defined as the reference series (all the original data were dimensionless and processed before analysis). The correlation coefficients between the reference series values and each comparison series were calculated, and the average value of the gray correlation coefficient was obtained, which was the gray correlation degree. The influence degree of each characteristic variable on hepatocyte toxicity was evaluated by comparing the gray correlation degrees.

#### 2.6.2 Orthogonal partial least squares analysis

OPLS, a special type of multiple linear regression model, was used to find the relationship between two matrices X and Y by considering orthogonal signal correction based on partial least squares regression (Liang et al., 2017; Liao et al., 2020). In this study, an OPLS model was constructed to characterize the correlation between the hepatotoxicity index IC_50_ and the chemical peaks. The peak area of each characteristic ion was used as the independent variable X, and the IC_50_ value was used as the dependent variable Y. In SIMCA 14.0.1 (Umetrics AB, Umea, Sweden), the VIP and regression coefficients were used to find the main characteristic components that were significantly correlated with hepatotoxicity.

#### 2.6.3 Back propagation artificial neural network analysis

The BP-ANN algorithm is a nonlinear mathematical model based on the structure of neural synaptic connections in the brain. The BP neural network is a kind of multilayer feedforward neural network trained by the error back propagation algorithm and has been one of the most widely used neural network models (Jiang et al., 2018; Shi et al., 2018). The BP neural network can connect the input and output parameters and can continuously modify the weights and biases of each layer through iterative learning to minimize the overall error of the output layer. To screen representative hepatotoxic components from different perspectives, we used MATLAB R2019b (Mathworks, Natick, NJ, United States) to build the BP-ANN model for the association of chromatographic peaks with hepatotoxicity IC_50_. The BP neural network was established using the characteristic peak area as the input layer neuron, the IC_50_ value as the output layer neuron, the hidden layer of one layer, and the hidden layer node number optimization as 10. Moreover, two parameters were used to evaluate the importance of the variables in the neural network.

MIV was considered to be one of the best indices for evaluating the correlation of variables in the neural network (Xu et al., 2013). The sign of the MIV value represents the direction of the correlation, and the absolute value reflects the importance of the impact. Sensitivity analysis was another important method for evaluating the connection weights in ANN models (Wang et al., 2017; Qiao et al., 2021). The contribution ratios of the characteristic peaks to the cytotoxicity index IC_50_ were calculated by connection weights. The Garson equation was applied to show the relative influence of the independent variables on the dependent variable. The equation was as follows:

$$P_{ac}\;=\;\frac{\sum_{b=1}^{N}\underset{}{\left(\frac{\left|w_{\mathrm{ab}}\right|}{\sum_{d=1}^{M}\left|w_{\mathrm{dj}}\right|}\left|e_{\mathrm{bv}}\right|\right)}}{\sum_{a=1}^{M}\left(\sum_{b=1}^{N}\left(\frac{\left|w_{\mathrm{ab}}\right|}{\sum_{d=1}^{M}\left|w_{\mathrm{dj}}\right|}\left|e_{\mathrm{bv}}\right|\right)\right)}$$

where *P* stands for the percentage influence of input neurons, *w* indicates the weight between input and hidden neurons, *e* indicates the weight between hidden and output neurons, *M* indicates the number of input neurons, *N* indicates the number of hidden neurons, and *v* indicates the number of output neurons.

## 3 Results

### 3.1 Characterization of chemical components in *Polygonum multiflorum*

Based on the literature summary and self-built compound library, the main components of PM are stilbenes and anthraquinones. In addition, PM includes flavonoids, lignans, dianthrones, phospholipids, and polysaccharides. Comparing the negative ion response with the positive ion response, the negative ion mode had more peaks and a much stronger response, so negative ion scan was selected for detection (Supplementary Figure S1). Moreover, the peak profiles of PM between raw and processed PM were basically consistent (Supplementary Figure S2), indicating that processing does not change the types of compounds in PM but the relative content of compounds. Considering the differences in the chemical composition of PM from different batches and origins, a mixed sample was chosen for qualitative analysis. The chromatographic column, mobile phase, elution conditions, and MS conditions were further optimized. A total of 112 components were detected and preliminarily identified through self-built database matching, comparison with standard products and the literature, and fragment ion deduction (Table 2). These tentative compounds could be classified into four types according to the structural characteristics, including 43 anthraquinones, 28 stilbene glycosides, 15 flavonoids, and 26 others.

**TABLE 2:** Ultraperformance liquid chromatography-quadrupole-time-of-flight-mass spectrometry identification results of chemical constituents of *Polygonum multiflorum*.

No	Observed RT (min)	Molecular formula	Component name	Observed *m/z*	Expected *m/z*	Mass error (ppm)	Fragment
1d	1.03	C_4_H_6_O_4_	Butanedioic acid	117.0190	117.0193	–3.22	71.0138; 59.0137; 55.0187
2d	1.05	C_6_H_8_O_4_	2,3-di-hydro-3,5-dihydroxy-6-methyl-4(*H*)-pyran-4-one	143.0350	143.0349	0.12	129.0187; 96.9687; 114.0557; 78.9591
3d	1.20	C_7_H_6_O_5_	Gallic acid	169.0147	169.0142	2.44	125.0246; 96.9687; 110.0254
4d	1.41	C_13_H_16_O_10_	Gallic acid-*O*-glucoside	331.0654	331.0665	–3.32	169.0107; 125.0221
5d	1.53	C_6_H_13_NO_2_	Leucine	130.0871	130.0868	2.31	88.0363; 85.0303
6d	2.25	C_6_H_8_O_7_	Citric acid	191.0201	191.0197	2.05	128.0355; 111.0086; 87.0088; 85.0294
7d	2.45	C_15_H_14_O_7_	Gallocatechin	305.0673	305.0666	2.09	213.1246; 241.0027; 125.0245; 96.9604
8d	3.16	C_13_H_16_O_9_	Protocatechuic acid-*O*-glucoside	315.0697	315.0716	–6.03	153.0177; 195.0297; 111.0094
9d	3.82	C_11_H_9_NO_2_	2-vinyl-1*H*-indole-3-carboxylic acid	186.0545	186.0555	–5.37	142.0658
10c	4.07	C_30_H_26_O_12_	Procyanidin B	577.1358	577.1351	1.10	289.0716; 559.1279; 451.1047; 407.0772; 125.0243
11c	4.22	C_15_H_10_O_7_	Quercetin	301.0355	301.0354	0.56	257.0455; 125.0243; 285.0397; 179.0243
12c	4.88	C_15_H_14_O_6_	Catechin	289.0721	289.0717	1.02	271.0553; 245.0812; 137.0244; 123.0450
13d	5.24	C_8_H_8_O_4_	Vanillic acid	167.0351	167.0344	4.19	137.0259; 123.0426
14d	5.83	C_7_H_6_O_2_	*P*-hydroxybenzaldehyde	121.0296	121.0295	0.99	93.0341
15b	6.54	C_21_H_22_O_11_	Rumejaposide D	449.1088	449.1089	–0.15	259.0612; 255.0660; 125.0242; 407.0769; 368.0900
16d	7.31	C_11_H_10_O_3_	Altechromone A	189.0560	189.0557	1.51	147.0448; 124.0157
17c	7.92	C_15_H_14_O_6_	Epicatechin	289.0718	289.0717	0.28	243.0660; 125.0244
18c	8.03	C_37_H_30_O_16_	3-*O*-galloyl-procyanidin B2	729.1465	729.1461	0.54	499.1267; 589.1452; 247.0619; 243.0660; 125.0244
19b	8.58	C_14_H_18_O_10_	2,3,4,6-tetrahy-droxyacetophenone-3-*O*-*β*-D-glucoside	345.0832	345.0827	1.44	182.0225; 242.0577; 287.0560; 125.0246; 96.9606
20c	8.67	C_30_H_26_O_12_	Isomer-Procyanidin B	577.1351	577.1351	-0.12	439.1056; 289.0715; 345.0818; 182.0225
21b	9.12	C_26_H_32_O_14_	Isomer-2,3,5,4′-tetrahydroxystilbene-2,3-di-*O-β*-D-glucopyranoside	567.1719	567.1719	0.01	405.1186; 387.1069; 241.0503; 281.0445
22d	9.61	C_17_H_20_O_9_	7-hydroxy-3,4-dimethylcoumarin-5-*O-β*-D-glucopyranoside	367.1029	367.1034	–1.45	243.0665; 225.0554; 109.0293
23b	9.61	C_20_H_22_O_9_	*Cis*-2,3,5,4′-tetrahydroxystilbene-2-*O-β*-D-glucoyranoside	405.1193	405.1191	0.52	243.0665; 189.0560; 137.0245; 93.0344
24c	10.01	C_35_H_34_O_15_	Polygonflavanol A	693.1821	693.1825	-0.61	549.1604; 287.0560; 259.0612; 125.0244; 241.0504
25a	10.91	C_15_H_12_O_4_	Emodin anthrone	255.0660	255.0662	–1.29	137.0241; 109.0288; 93.0345
26a	10.92	C_22_H_26_O_8_	1,3-dihydroxy-6,7-dimethylxanthone-1-*O-β*-D-glucopyranoside	417.1184	417.1555	–1.75	259.0609; 255.0659; 109.0288; 137.0242
27a	11.08	C_20_H_22_O_10_	6-methoxyl-2-acetyl-3-methyl-1,4-naphthoquinone-8-*O-β*-D-glucopyranoside	421.1137	421.1140	–0.73	407.0767; 259.0610; 255.0660; 213.0555
28c	11.11	C_44_H_34_O_20_	3,3′-di-*O*-galloyl-procyanidin B2	881.1582	881.1571	1.24	729.1451; 513.1201; 407.0767; 273.0391
29a	11.18	C_16_H_10_O_7_	Carboxyl emodin	313.0344	313.0348	–1.28	269.0433; 243.0634; 169.0107
30c	11.65	C_22_H_18_O_10_	Epicatechin-3-*O*-gallate	441.0828	441.0827	0.16	289.0714; 169.0145; 125.0245
31c	11.75	C_15_H_10_O_6_	Kaempferol	285.0402	285.0404	–1.08	193.0142; 125.0245
32d	11.89	C_28_H_38_O_13_	(+)-lyoniresinol-3-*O-β*-D-glucopyranoside	581.2239	581.2240	–0.15	549.1606; 521.1300; 387.1072; 253.0081
33b	12.22	C_22_H_24_O_10_	2,3,5,4′-tetrahydroxystilbene-2-*O*-(6-*O*-acetyl)-*β*-D-glucopyranoside	447.1288	447.1296	-1.88	259.0608; 227.0713; 185.0608
34b	12.70	C_26_H_32_O_14_	2,3,5,4′-tetrahydroxystilbene-2,3-di-*O-β*-D-glucopyranoside	567.1724	567.1719	0.84	405.1179; 269.0455; 243.0664; 225.0553
35a	13.16	C_16_H_12_O_6_	Fallacinol	299.0558	299.0561	–1.15	286.0480; 253.0495; 161.0243; 179.0354
36b	13.21	C_26_H_34_O_11_	*β*-D-glucoside,4-[2,3-dihydro-3-(hydroxymethyl)-5-(3-hydroxypropyl)-7-methoxy-2-yl]-2-methoxypheny	521.2054	521.2023	5.95	359.1455; 313.1039; 243.0634
37a	13.26	C_21_H_22_O_11_	Isomer-rumejaposide D	449.1090	449.1089	0.19	379.0815; 169.0145; 165.0558; 286.0480
38b	13.51	C_60_H_66_O_27_	Multiflorumiside L/K	1,217.3710	1,217.3718	–0.68	811.2458; 646.1675; 243.0665; 405.1189
39b	13.52	C_20_H_22_O_9_	*Trans*-2,3,5,4′-tetrahydroxystilbene-2-*O*-*β*-D-glucopyranoside	405.1191	405.1191	-0.08	243.0665; 225.0554; 109.0293; 215.0713
40a	14.08	C_47_H_46_O_22_	PM 12-13	961.2381	961.2408	–2.79	693.1812; 503.1164; 555.1137; 393.0609; 839.2375
41b	14.47	C_19_H_20_O_8_	2,3,5,4′-tetrahydroxystilbene-2-*O-β*-D-xyloside	375.1080	375.1085	–1.54	243.0665; 225.0553; 109.0291
42b	14.61	C_27_H_26_O_13_	2,3,5,4′-tetrahydroxystilbene-2-*O-β*-D-(2-*O*-monogalloyl esters)-glucopyranoside	557.1310	557.1301	1.70	243.0666; 241.0504; 313.0567; 405.1189; 125.0243
43c	15.54	C_21_H_20_O_12_	Quercetin 3-*β*-D-glucopyranoside	463.0860	463.0882	–4.81	405.1171; 303.0514; 379.0815; 269.0456
44c	15.60	C_15_H_12_O_7_	Dihydroquercetin	303.0477	303.0505	–9.24	153.0177; 125.0221
45d	15.84	C_17_H_17_NO_4_	*Trans*-*N*-caffeoyltyramine	298.1084	298.1084	–0.28	169.0836; 227.0710; 135.0450
46b	16.45	C_27_H_26_O_13_	2,3,5,4′-tetrahydroxystilbene-2-*O-β*-D-(3-*O*-monogalloyl esters)-glucopyranoside	557.1310	557.1301	1.77	243.0664; 313.0567; 405.1180; 467.1097; 125.0244
47b	16.59	C_27_H_26_O_12_	2,3,5,4′-tetrahydroxystilbene-2-*O-β*-D-(2″-*O*-galloyl)-glucopyranoside	541.1355	541.1352	0.65	243.0664; 313.0567; 169.0145; 405.1180; 467.1097
48b	16.82	C_14_H_12_O_3_	Resveratrol	227.0716	227.0713	1.04	181.0648; 143.0502; 135.0446; 117.0344
49b	17.53	C_27_H_26_O_12_	*β*-Glucopyranoside, 3-hydroxy-5-[(1*E*)-2-(4-hydroxyphenyl)ethenyl]phenyl, 2-(3,4,5-trihydroxybenzoate)	541.1351	541.1352	–0.05	485.1242; 313.0564; 169.0145
50d	18.00	C_19_H_22_O_9_	7-acetyl-3,8-dihydroxy-6-methyl-1-naphthyl-*β*-D-glucopyranoside	393.1190	393.1191	–0.24	273.0767; 231.0665; 295.0583; 161.0245
51b	18.47	C_22_H_24_O_10_	Polygonibene D	447.1292	447.1296	–1.06	255.0660; 243.0664; 241.0502
52c	19.63	C_23_H_24_O_12_	Tricin 7-*O-β*-D-glucoside	491.1191	491.1195	–0.74	269.0451; 313.0553; 148.0526; 355.0447; 439.0652
53d	19.75	C_18_H_19_NO_4_	*N*-*trans*-feruloyltyramine	312.1240	312.1241	–0.29	274.0120; 269.0451; 178.0516; 148.0526; 123.0452
54a	19.94	C_22_H_26_O_10_	Torachrysone-8-*O*-(6′-*O*-acetyl)-*β*-D-glucopyranoside	449.1447	449.1453	–1.39	393.0615; 274.0120; 245.0815; 230.0584; 349.0699
55b	20.13	C_29_H_28_O_12_	Tetrahydroxystilbene-*O*-(caffeoyl)-glucopyranoside	567.1498	567.1503	–0.88	243.0634; 405.1207
56b	20.40	C_20_H_22_O_8_	Polydatin	389.1237	389.1242	–1.19	283.0608; 227.0711
57d	20.50	C_19_H_21_NO_5_	*N*-trans-feruloyl-3-methyldopamine	342.1341	342.1347	–1.61	313.0546; 227.0711; 255.0656; 148.0524
58b	20.75	C_21_H_24_O_8_	Desoxyrhaponticin	403.1392	403.1398	–1.62	349.0708; 269.0453; 225.0552; 151.0037
59b	21.65	C_30_H_30_O_12_	Polygonibene G	581.1662	581.1665	–0.39	419.1125; 295.0600; 389.1015; 125.0244
60a	21.85	C_21_H_20_O_10_	Aloe-emodin-3-(hydroxymethyl)-*O-β*-D-glucopyranoside	431.0987	431.0983	0.70	240.0428; 325.0707; 268.0372; 299.0561
61a	22.11	C_23_H_22_O_11_	Emodin-8-*O*-(6′-*O*-acetyl)-*β*-D-glucopyranoside	473.1093	473.1089	0.88	269.0459; 225.0558
62b	22.60	C_29_H_28_O_11_	2,3,5,4′-tetrahydroxystilbene-2-*O-β*-D-(2″-O-coumaroyl)-glucoside	551.1545	551.1553	–1.45	389.1003; 241.9957; 405.1207
63b	22.90	C_30_H_30_O_12_	Polygonibene E	581.1669	581.1665	0.83	405.1184; 243.0663; 256.0375
64a	22.95	C_25_H_32_O_13_	Polygonimitin E	539.1765	539.1770	–0.91	405.1184; 243.0663; 256.0375; 489.1212; 175.0400
65d	23.09	C_36_H_36_N_2_O_8_	Cannabisin D	623.2389	623.2399	–1.66	389.1026; 245.0814; 225.0555
66a	23.73	C_20_H_24_O_9_	Torachrysone-8-*O-β*-D-glucopyranoside	407.1346	407.1347	–0.39	245.0820; 230.0587; 215.0352
67a	24.05	C_16_H_12_O_6_	Citreorosein-8-methyl ether	299.0556	299.0561	–1.80	255.0656; 243.0660; 213.0552; 160.0162
68a	24.74	C_16_H_12_O_5_	Emodin-8-methyl ether	283.0611	283.0612	–0.51	240.0426; 175.0400; 145.0296
69c	24.96	C_21_H_20_O_11_	Quercetin-3-*O*-rhamnoside	447.0931	447.0933	–0.45	285.0399; 313.0546; 337.0788; 361.0725; 245.0810
70a	25.40	C_15_H_10_O_5_	Isomer-emodin	269.0458	269.0455	0.26	93.03439; 185.0607
71a	25.62	C_21_H_20_O_10_	Emodin-8-*O-β*-D-glucopyranoside	431.0985	431.0983	0.34	269.0459; 225.0559
72a	26.09	C_45_H_44_O_21_	PM 5	919.2315	919.2302	1.35	875.2393; 713.1859; 458.1210; 416.1108
73a	27.03	C_45_H_44_O_21_	Isomer-PM 5	919.2303	919.2302	0.07	875.2387; 713.1860; 458.1215
74d	27.22	C_36_H_36_N_2_O_8_	(+)-Grossamide	623.2395	623.2399	–0.57	269.0458; 243.0660; 416.1106
75c	27.32	C_17_H_14_O_7_	Tricin	329.0660	329.0666	–2.13	243.0660; 313.0484; 161.0246; 254.0583
76a	27.72	C_22_H_22_O_10_	Physcion-1-*O-β*-D-glucopyranoside	445.1135	445.1140	–1.15	283.0611; 240.0426; 145.0295; 387.0501
77a	28.11	C_15_H_10_O_6_	Citreorosein	285.0410	285.0404	1.78	257.0455; 241.0503; 224.0477; 195.0452; 183.0452
78a	28.46	C_17_H_14_O_5_	1,6-dimethyl ether-emodin	297.0765	297.0768	–1.06	283.0612; 269.0458; 240.0428
79a	28.51	C_22_H_22_O_10_	Physcion-8-*O-β*-D-glucopyranoside	445.1138	445.1140	–0.40	283.0612; 240.0428; 225.0552; 148.0529
80a	28.80	C_22_H_26_O_10_	Isomer-torachrysone-8-*O*-(6′-*O*-acetyl)-*β*-D-glucopyranoside	449.1450	449.1453	–0.66	255.0658; 245.0815; 230.0584; 359.0909; 159.0445
81a	29.00	C_21_H_20_O_11_	Citreorosein-*O*-glucoside	447.0931	447.0933	-0.50	243.0659; 211.1340; 329.2333; 125.0245
82a	30.92	C_16_H_12_O_5_	Isomer-physcion	283.0611	283.0612	–0.39	269.0454; 239.0326
83a	31.52	C_45_H_46_O_19_	PM 26-27	889.2553	889.2561	–0.80	847.2462; 701.1841; 458.1212; 416.1108; 254.0580
84a	33.16	C_17_H_12_O_6_	2-Acetyl-emodin	311.0562	311.0561	0.15	283.0606; 269.0457; 240.0429
85a	33.40	C_37_H_34_O_13_	Polygonumnolide E	685.1922	685.1927	–0.73	671.1752; 416.1109; 309.1735; 254.0586
86a	34.64	C_15_H_10_O_4_	Chrysophanol	253.0498	253.05	–1.19	225.0545
87a	34.74	C_15_H_10_O_5_	Emodin	269.0459	269.0455	1.32	225.0560; 241.0505; 197.0608
88a	36.87	C_30_H_22_O_8_	*Trans/cis*-emodin dianthrones	509.1245	509.1242	0.58	254.0582; 225.0545
89a	37.13	C_15_H_10_O_6_	Lunatin	285.0404	285.0404	–0.26	269.0457; 241.0501; 199.1704
90a	37.17	C_16_H_12_O_5_	Physcion	283.0609	283.0612	–0.96	269.0456; 256.0362; 240.0422
91a	38.69	C_31_H_24_O_8_	*Trans/cis*-emodin-physcion dianthrones	523.1395	523.1398	–0.68	254.0583
92a	40.37	C_32_H_26_O_8_	*Trans/cis*-physcion dianthrones	537.1541	537.1555	–2.60	243.0661; 437.3076; 339.1998
93d	41.55	C_16_H_32_O_2_	Tetradecanoic acid ethyl ester	255.2333	255.2329	1.31	205.1602; 96.9602
94d	42.22	C_17_H_34_O_2_	Hexadecanoic acid methyl ester	269.2485	269.2486	–0.47	177.9736; 129.9760; 221.0857
95d	42.92	C_18_H_36_O_2_	Hexadecanoic acid ethyl ester	283.2645	283.2642	0.75	183.0122; 99.0194; 163.1127
96d	43.13	C_20_H_38_O_2_	Ethyl oleate	309.2796	309.2799	–1.11	163.1127; 177.1283; 223.0358; 227.2015
97d	43.47	C_19_H_38_O2	Octadecanoic acid methyl ester	297.2796	297.2799	–0.89	241.0502; 119.9469
98d	44.01	C_20_H_40_O_2_	Octadecanoic acid ethyl ester	311.2956	311.2955	0.15	229.1596; 163.1130; 130.9451
99b	12.37; 13.19	C_41_H_46_O_19_	(unknown) Dimer of stilbene glycoside	841.2562	841.2561	0.12	647.1770; 485.1239; 259.0608; 227.0713; 125.0243
100b	15.12; 16.18; 17.64; 18.56	C_40_H_42_O_18_	(Isomer) Multiflorumiside A1/B1	809.2308	809.2298	1.15	647.1773; 719.1815; 485.1239; 467.1109; 267.0651
101b	19.90; 20.67	C_27_H_24_O_13_	Polygonumoside A/B	555.1154	555.1144	1.86	393.0615; 274.0120; 245.0815; 230.0584; 349.0699
102b	21.48; 22.52	C_40_H_42_O_18_	Polygonibene A/B/C	809.2295	809.2298	–0.44	647.1766; 485.1236; 255.0657; 405.1174; 125.0244
103a	24.52; 29.27	C_23_H_22_O_11_	Isomer-emodin-8-*O*-(6′-*O*-acetyl)-*β*-D-glucopyranoside	473.1091	473.1089	0.26	269.0454; 225.0552; 167.0349
104a	25.27; 26.92; 27.67; 28.47	C_46_H_46_O_21_	PM 22-25	933.2457	933.2459	–0.15	889.2548; 809.2265; 703.1669; 458.1210; 283.0611
105a	27.37; 30.01	C_42_H_42_O_18_	PM 1-4	833.2306	833.2298	0.90	671.1764; 431.0980; 416.1110; 175.0398; 254.0583
106a	28.64; 29.77; 30.41	C_39_H_34_O_16_	PM 14-17	757.1769	757.1774	–0.68	713.1868; 458.1210; 269.0458; 225.0552
107a	29.52; 30.89; 31.57	C_43_H_44_O_18_	Polygonumnolides B1-B3	847.2463	847.2455	0.99	707.1738; 685.1909; 416.1108; 283.0607; 240.0428
108a	30.86; 31.06; 31.24; 31.49	C_40_H_36_O_16_	PM 30-33	771.1927	771.1931	–0.51	458.1212; 398.0987; 416.1109; 285.0400; 254.0580
109a	32.04; 32.27; 33.51; 33.99	C_36_H_32_O_13_	Polygonumnolides C1-C4	671.1775	671.1770	0.76	265.1480; 458.1207; 553.1048; 416.1111; 254.0586
110a	33.41; 33.72; 34.76; 34.92	C_37_H_34_O_13_	Polygonumnolides A1-A4	685.1931	685.1927	0.66	671.1752; 416.1109; 309.1735; 254.0586
111b	4.58; 5.69; 6.57; 8.04	C_40_H_44_O_19_	(Isomer) Polygonumoside C/D	827.2403	827.2404	–0.07	665.1867; 467.1116; 269.0455; 225.0542; 131.0827
112b	5.91; 10.32; 10.38; 12.75; 13.50	C_40_H_44_O_18_	Multiflorumiside A-I	811.2442	811.2455	–1.64	649.1914; 487.1372; 405.1182; 243.0662; 225.0553

a: Anthraquinones and derivatives. b: Stilbenes and derivatives. c: Flavonoids and derivatives. d: Others. The names of PM 1-4, PM 5, PM 14-17, PM 22-25, and PM 26-27 were from Yang, J. B. (2019). Journal of pharmaceutical and biomedical analysis, 172, 149-166.

### 3.2 Metabolomics analysis of raw and processed *Polygonum multiflorum*

**FIGURE 2:**
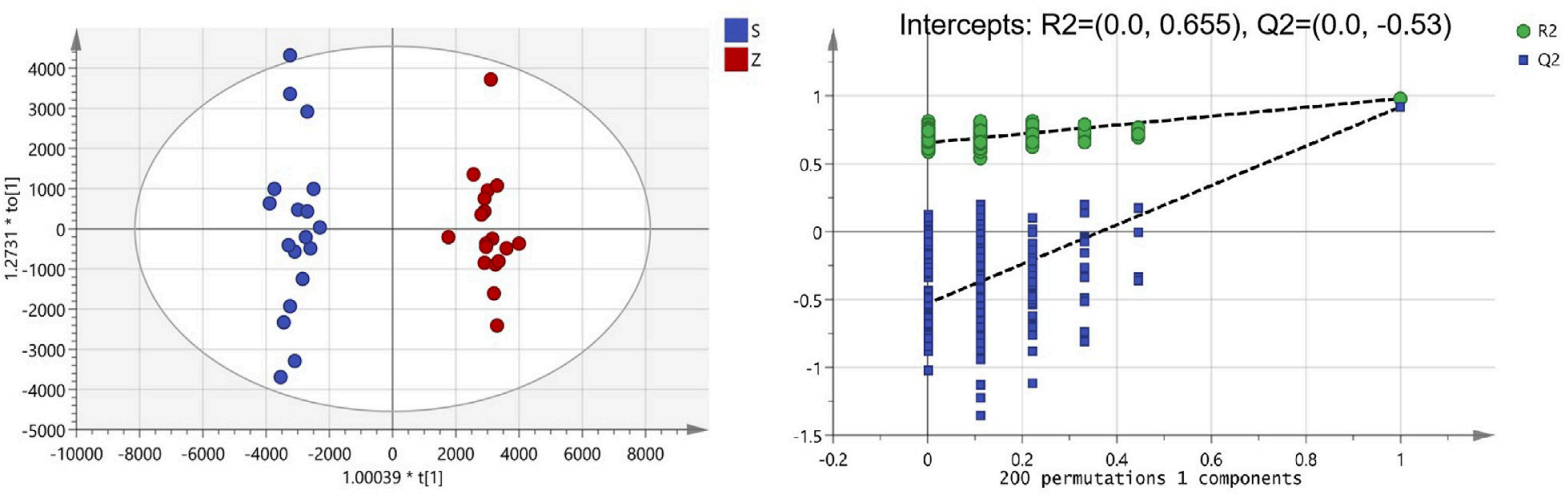
Orthogonal partial least squares analysis-DA score chart and permutation test analysis of *Polygonum multiflorum* (PM) samples (S: raw PM; Z: processed PM).

The clinical use of PM usually includes both raw and processed PM. Previous studies have shown that the chemical composition of processed PM may change compared with that of raw PM, which may lead to a change in the pharmacological effects. For a fact, various studies have also shown that the toxicity of PM was significantly reduced after processing, which may be due to the significant reduction of toxic ingredients. To date, few studies have been performed to fully clarify the compositional changes in PM after treatment. Here, UPLC-Q-TOF-MS analysis combined with multivariate statistical analysis was used to distinguish between raw and processed PM. The PCA graph shows that the QC samples were closely clustered, indicating that the LC-MS analysis system was credibly reproducible and stable during the testing period. As seen from the PCA plots (Supplementary Figure S3), the raw PM and manufactured PM samples were able to be obviously separated and gathered separately. To further screen out the latent variables for distinguishing between raw and processed PM, OPLS-DA analysis was performed. The R2Y and Q2 of the OPLS-DA model were 0.98 and 0.92, respectively, which indicated excellent fitness and reliability. From the results (Figure 2), it was evident that the raw and processed PM were significantly differentiated under the supervised model. There was no overfitting in the OPLS-DA model by 200-times permutation tests, as shown in Figure 2. Furthermore, with VIP > 1.5, univariate statistical analysis *p* < 0.5, and fold change < 0.5, 126 differential characteristic ions were screened for significant reduction after preparing PM. Combined with the results of the abovementioned component analysis, 13 potential compounds were identified after excluding the interfering fragments and confirming the molecular ions. The results are shown in Table 3.

**TABLE 3:** Detailed information of 13 different compounds between raw and processed *Polygonum multiflorum* .

Compounds	Rt-*m/z* (Da)	VIP	*p*-value	FC-value
Catechin	4.88_289.0716	11.03	1.50E^−6^	0.286
Epicatechin	7.89_290.0786n	4.08	4.60E^−4^	0.397
Torachrysone-8-*O-β*-D-glucopyranoside	23.71_408.1413n	9.99	1.00E^−9^	0.225
7-acetyl-3,8-dihydroxy-6-methyl-1-naphthyl-*β*-D-glucopyranoside	17.97_393.1173	3.48	2.42E^−10^	0.137
Epicatechin-3-*O*-gallate	11.59_442.0917n	7.44	9.32E^−5^	0.328
Emodin-8-*O-β*-D-glucopyranoside	25.61_431.2031	2.47	1.75E^−8^	0.441
2,3,5,4′-tetrahydroxystilbene-2-*O-β*-D-(2-*O*-monogalloylesters)-glucopyranoside	14.58_558.1371n	13.36	5.97E^−5^	0.359
polygonibene E	22.91_582.1726n	5.56	2.50E^−7^	0.366
Polygonumnolides C1-C4	32.40_671.1733	1.83	5.55E^−3^	0.380
Polygonumnolides A1-A4	34.95_685.1887	2.06	3.15E^−3^	0.366
PM 14-17	30.37_758.1799n	1.59	2.04E^−3^	0.157
PM 22-25	27.64_933.2410	1.86	7.60E^−5^	0.186
PM 5	27.10_920.2342n	1.77	1.23E^−4^	0.180

### 3.3 Pseudotargeted spectrum construction of discriminant metabolites

**FIGURE 3:**
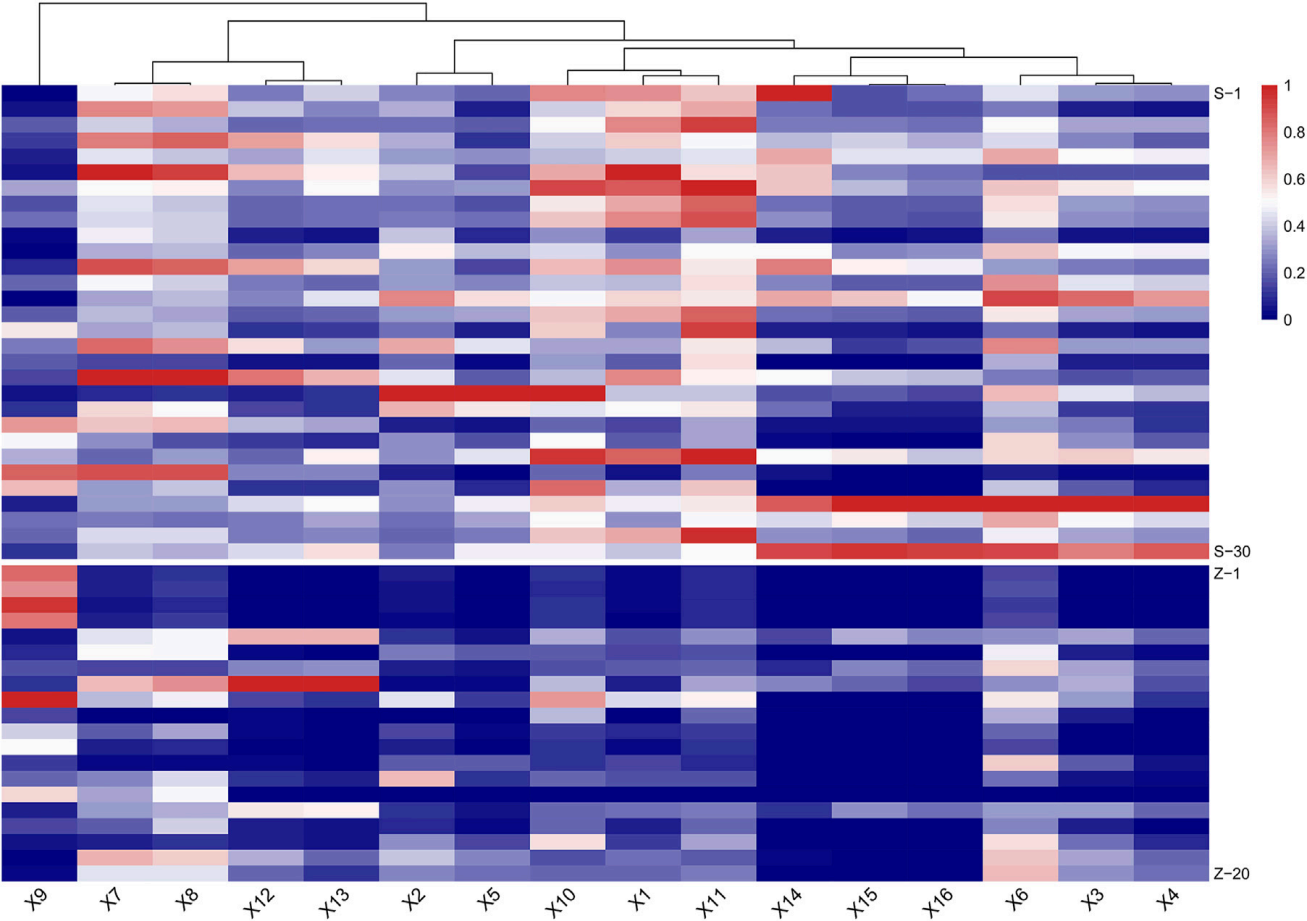
Heat map of semiquantitative analysis of 16 target compounds between raw and processed *Polygonum multiflorum* (PM) (S: raw PM; Z: processed PM).

In MRM-based absolute quantification, calibration curves were often drawn for real compounds based on the conversion of the corresponding peak area into the content. However, absolute quantification usually cannot be achieved owing to the limitations of the standards, and the relative content between different groups can be compared by peak area. In consideration of the potential toxic dianthrone components identified in our previous studies and dianthrone aglycon hydrolyzed in acidic gastric juice *in vivo*, three nuclear parents of dianthrones were summarized (Li et al., 2020; Wang et al., 2021; Yang et al., 2021). Combined with the 13 differential metabolites obtained from the metabonomics analysis, UPLC-qqq-MS/MS semiquantitative profiles were further established. By optimizing the MRM ion pair and CV and CE values, semiquantitative mass spectra of the 16 compounds were constructed. The results are listed below. This method was successfully applied to 30 batches of raw PM and 20 batches of processed PM, and the standardized peak area heatmap is shown in Figure 3.

At last, a methodological investigation on the established scheduled MRM method, including specificity, linearity, precision, repeatability, and stability, was conducted. The 16 target compounds showed great specificity (Supplementary Figure S4). Among the 16 target analytes, linearity was good in the range of 0.5–3 µl injection with R > 0.98. Precision and repeatability results showed that the RSD values of all 16 compounds were less than 15%. For stability within 30 h, the RSD values ranged between 1.53% and 14.7% for all components.

### 3.4 Hepatotoxicity evaluation of *Polygonum multiflorum*

**FIGURE 4:**
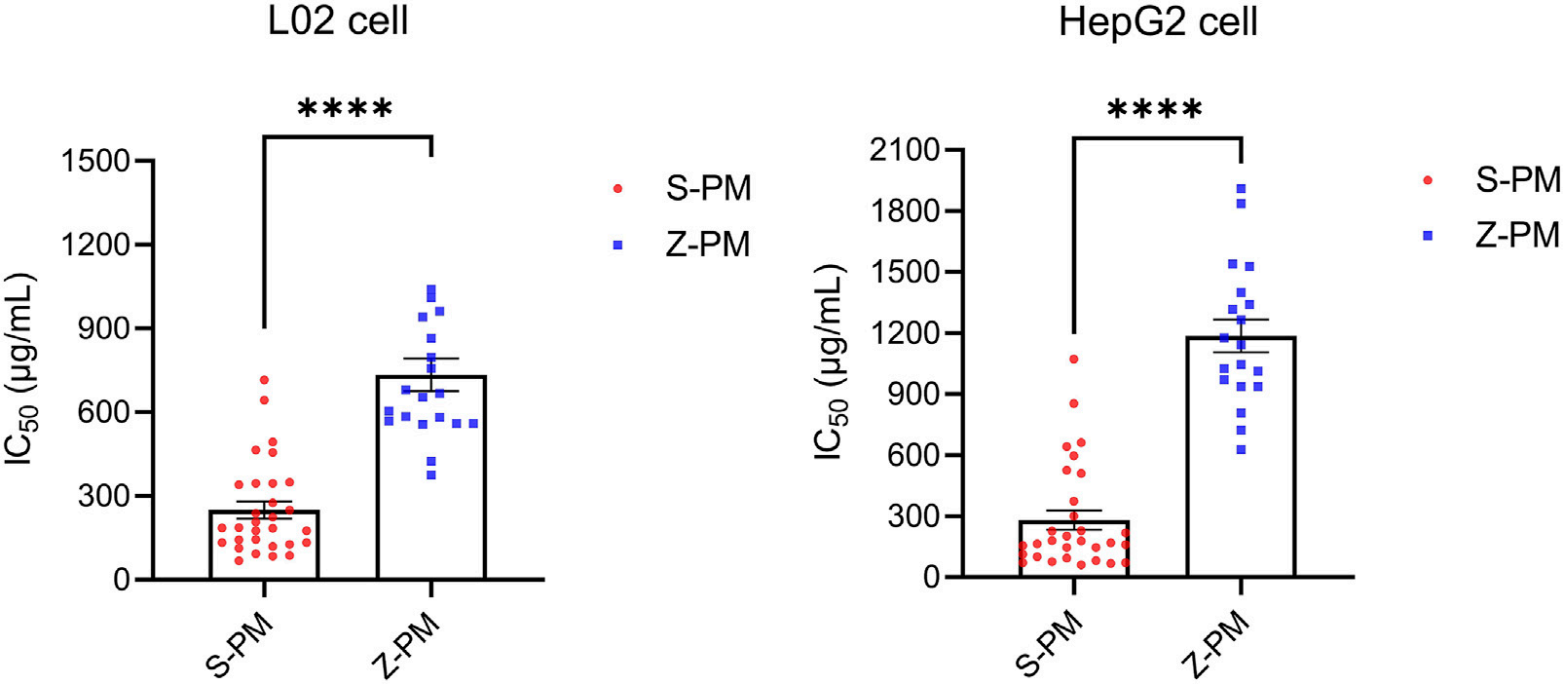
Statistical analysis of IC_50_ values of 50 batches of raw and processed *Polygonum multiflorum* on two kinds of hepatocytes (*p* < 0.0001, ****).

It is necessary to evaluate hepatotoxicity *in vitro*, but sometimes a cellular model does not provide an accurate and comprehensive assessment of the hepatotoxicity of TCM. In this study, two commonly used hepatocyte models were chosen, L02 and HepG2, to comprehensively estimate the hepatotoxicity of raw and processed PM extracts. The IC_50_ values for the raw and processed PM are shown in Supplementary Table S3. From Figure 4, the mean IC_50_ values of PM in both types of hepatocytes increased significantly after processing (*p* < 0.0001, ****), indicating the basic theories of processing detoxification. In specific, 30 batches of raw PM had an average IC_50_ value of 250 μg/mL in L02 cells and 281 μg/mL in HepG2 cells. However, 20 batches of processed PM had an average IC_50_ value of 735 μg/ml in L02 cells and 1,185 μg/ml in HepG2 cells.

### 3.5 Results of spectrum–effect relationship

#### 3.5.1 Gray relational analysis results

The relationship between chromatographic peaks and hepatotoxicity effect was established by the GRA model. The degree of correlation between each chromatographic peak and hepatocyte toxicity is detailed in Table 4. The results showed that the gray relational degree between all 16 chromatographic peaks and the 1/IC_50_ of L02 cells was between 0.718 and 0.826. The correlation between the 16 peaks and the 1/IC_50_ of HepG2 cells was between 0.618 and 0.816. These results indicated that the 16 chromatographic peaks were closely correlated with hepatocyte toxicity. In total, dianthrone components X7, X8, X9, X12, X13, X14, X15, and X16; anthraquinone glycoside components X3, X4, and X6; stilbene glycosides X10 and X11; and flavanol compounds X1, X2, and X5 were all associated with hepatotoxicity in hepatocytes, which may synergistically contribute to the hepatotoxicity of PM.

**TABLE 4:** Correlation degree between peak areas of 16 targeted compounds and hepatotoxicity.

Compound	GRA (correlation)		OPLS (R value)	
	L02	HepG2	L02	HepG2
X1	0.763	0.754	0.873	0.478
X2	0.818	0.778	–0.285	0.061
X3	0.806	0.813	0.193	–0.055
X4	0.804	0.816	–0.062	–0.150
X5	0.825	0.814	–0.515	–0.510
X6	0.826	0.776	–0.387	–0.210
X7	0.755	0.718	–0.443	–0.222
X8	0.750	0.707	0.160	0.070
X9	0.718	0.681	–0.101	–0.190
X10	0.800	0.776	–0.266	–0.318
X11	0.786	0.769	–0.191	–0.062
X12	0.733	0.715	0.504	0.506
X13	0.744	0.755	0.239	0.198
X14	0.772	0.779	–0.597	–0.440
X15	0.750	0.774	0.048	–0.121
X16	0.755	0.775	–0.272	–0.252

#### 3.5.2 Orthogonal partial least squares analysis results

**FIGURE 5:**
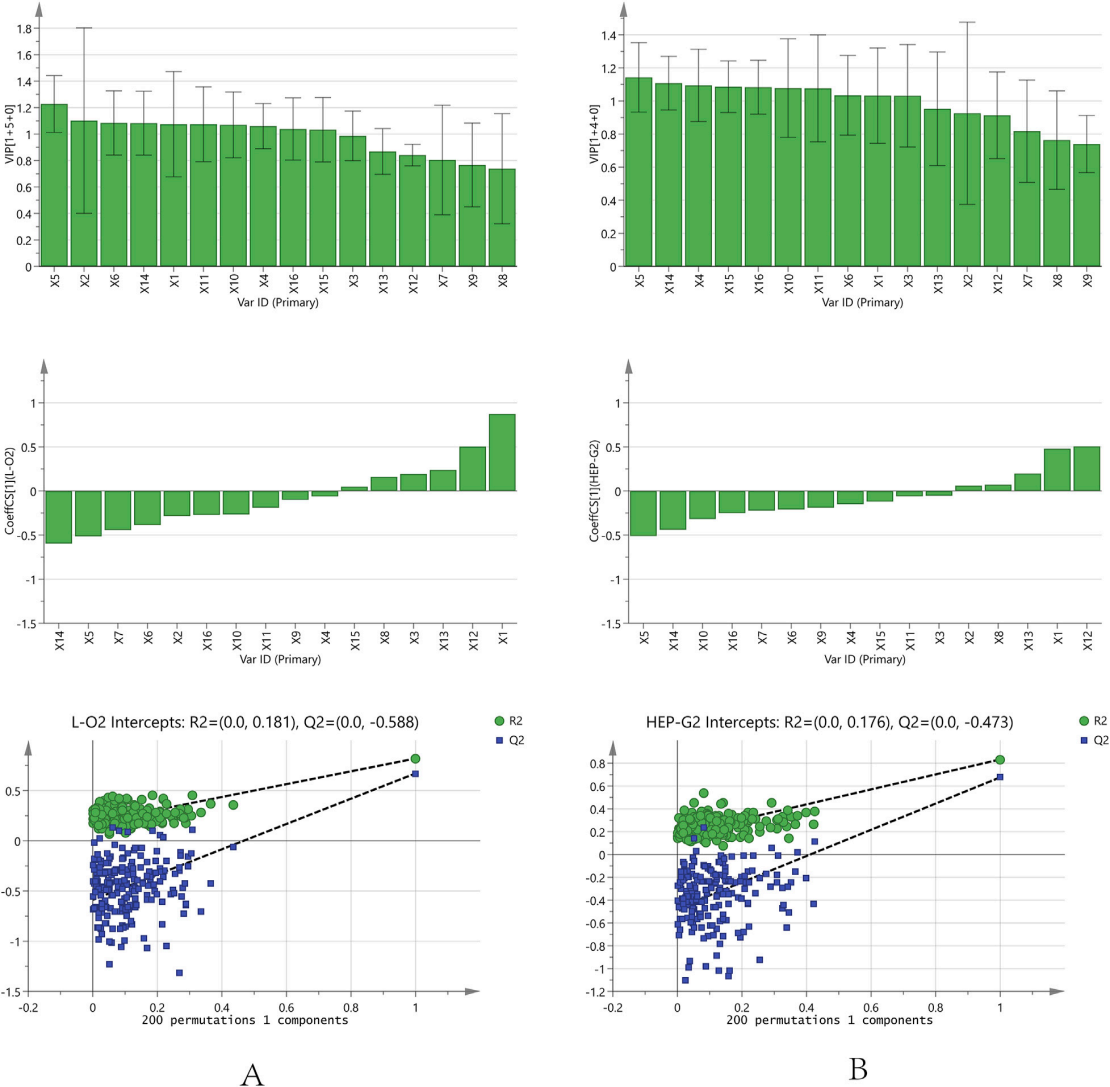
Orthogonal partial least squares analysis model correlation analysis and permutation test analysis results [**(A)**: L02 cell; **(B)**: HepG2 cell].

OPLS analysis was conducted using an orthogonalized multiple linear regression model. In this study, an OPLS model was built to analyze the correlation between the chromatographic peaks of 16 compounds and the IC_50_ of L02 and HepG2 hepatocytes (Figure 5). For L02 hepatocytes, the constructed model parameters R2X, R2Y, and Q2 were 0.94, 0.82, and 0.67, respectively. The permutation test was performed without overfitting. The results showed that the VIP values of all compounds were greater than 0.7. Combined with the correlation coefficient of less than 0.1, compounds X14, X5, X6, X7, X9, X2, X16, X10, and X11 were screened out. For HepG2 hepatocytes, the model parameters of R2X, R2Y, and Q2 were 0.93, 0.83, and 0.68, respectively, and the model had no overfitting. X5, X14, X10, X16, X7, X6, X9, X4, and X15 were highlighted with correlation coefficients less than −0.1 and VIP values greater than 0.7. For further analysis, the common significant components screened by both models were dianthrone components X7, X9, X14, and X16; anthraquinone glycoside X6; stilbene glycoside X10; and flavanol X5. These components may be of more prominent importance in the multicomponent synergistic hepatotoxicity of PM.

#### 3.5.3 Back propagation artificial neural network results

**FIGURE 6:**
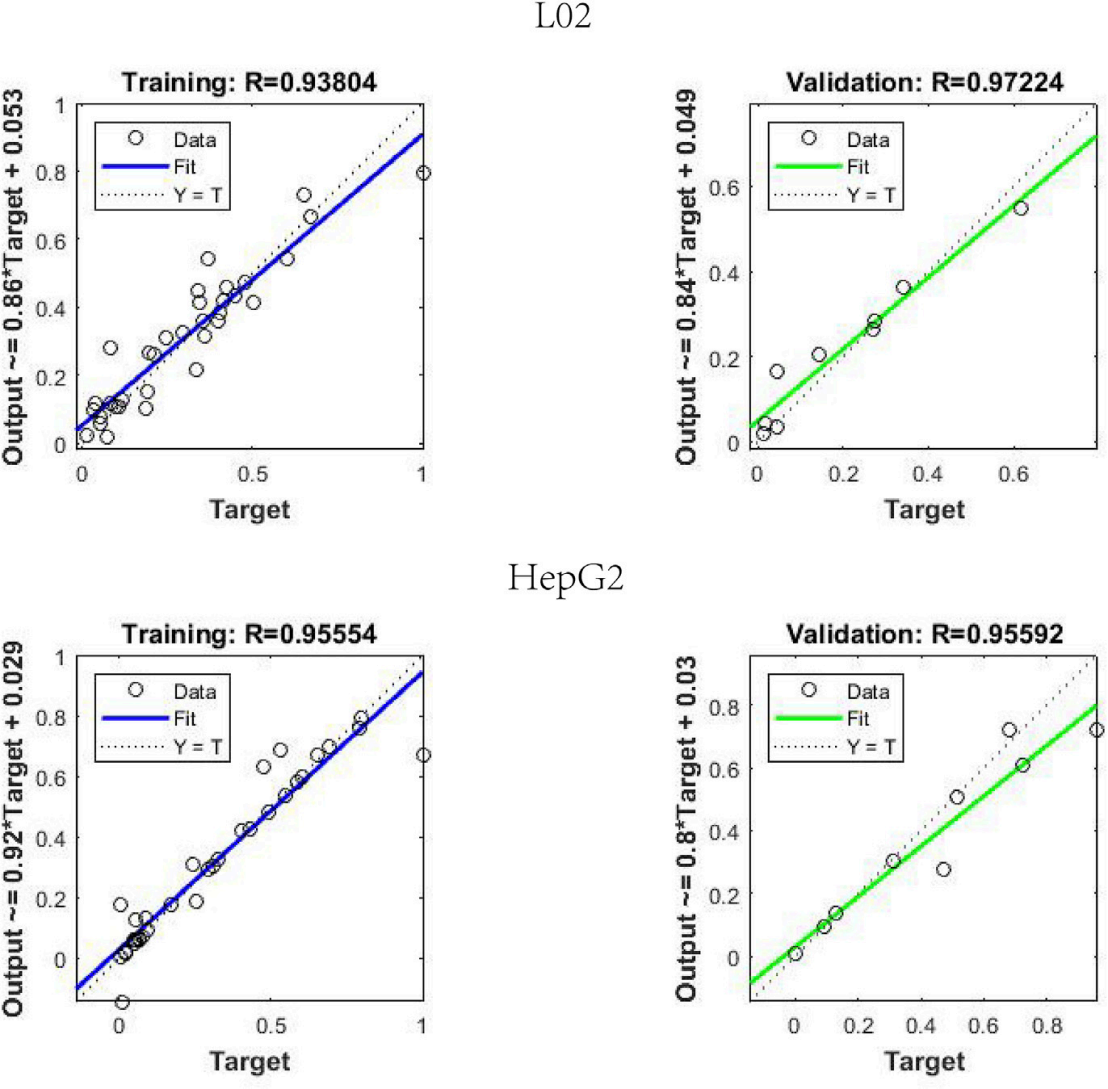
Regression R value results in back propagation artificial neural network neural network model.

BP-ANN is a multilayer network that uses an error back propagation algorithm for constant adjustment of weights. In this experiment, a simple 3-layer BP-ANN neural network was modeled with an input layer, one hidden layer, and an output layer. The fitting degree of the model was evaluated using the mean square error (MSE) and regression R value. In the model, 80% of the random sample data were taken as the training set, and 20% of the sample data were used as the validation set. The results (Figure 6) demonstrated that for L02 cells, the established neural network model, where the R of the training and validation datasets reached 0.9380 and 0.9722, the MSE of the training and validation datasets reached 0.006 and 0.0027, respectively. For HepG2 cells, the R and MSE of the training and validation datasets on the model were 0.9555 and 0.9559, 0.0068 and 0.0125, respectively.

As a result of the sensitivity analysis, the 16 compounds all had relatively average contributions (*p* value); the L02 cells ranged from 4.07 to 8.50, and the HepG2 cells ranged from 4.08 to 9.43. The specific data are shown in Table 5, and the 16 compounds had a relatively average influence on hepatocyte toxicity. The hepatotoxicity caused by PM may be due to the synergistic result of multiple components. Furthermore, the average influence value (MIV) of the input variables in the network was used to assess the importance of different variables in the BP-ANN model. Variables negatively correlated with the IC_50_ values were sieved out. For L02 cells, the screened components were X7, X6, X10, X4, X15, X9, X2, X14, and X16. For HepG2 cells, X7, X4, X11, X13, X6, and X14 were selected. In summary, the common components screened were dianthrone components X7 and X14 and anthraquinone glycosides X4 and X6. These components may be of great significance as potential hepatotoxic components in PM.

**TABLE 5:** Sensitivity analysis and MIV analysis results of 16 compounds in back propagation artificial neural network model.

Compound	*p* Value		MIV	
	L02	HepG2	L02	HepG2
X1	4.07	7.57	0.009	0.015
X2	5.01	9.43	-0.029	0.004
X3	7.29	5.69	0.105	0.029
X4	7.03	6.51	-0.046	-0.131
X5	6.60	4.08	0.002	0.062
X6	7.35	6.05	-0.089	-0.023
X7	6.40	7.40	-0.161	-0.247
X8	8.50	6.19	0.086	0.145
X9	4.99	6.21	-0.038	0.020
X10	7.61	6.63	-0.055	0.033
X11	5.77	6.19	0.022	-0.103
X12	8.12	5.91	0.072	0.178
X13	5.47	6.07	0.002	-0.102
X14	4.88	5.32	-0.015	-0.006
X15	5.70	4.95	-0.040	0.004
X16	5.22	5.79	-0.013	0.032

For the key characteristic components screened out using the above three models, the intersection of these components included X6, X7, and X14. It was thought that they may play a more significant role in liver injury caused by PM and could be used as toxicity markers of hepatotoxicity. We acknowledge that PM has complex chemical components and that its hepatotoxicity may be the result of the synergistic action of various components. The 16 components screened above all contained a degree of hepatotoxicity. Moreover, there were many potentially hepatotoxic compounds that we had not discovered and identified that need to be further explored and verified.

## 4 Discussion

As an invaluable treasure of Chinese civilization, Chinese herbal medicine has the characteristics of multiple components, multiple targets, and multiple pathways. Many previous studies have explored the material basis of PM-induced hepatotoxicity through different methods. The results showed that it was not one type of compound that was responsible for hepatotoxicity in PM, which reflected the complexity and holistic nature of TCM. The hepatotoxicity may be a synergistic effect caused by multiple components acting on multiple targets leading to the toxicity result. In this study, MS fingerprints were combined with pharmacological toxicity to target potential hepatotoxic compounds in PM. Sixteen compounds were found to be potentially associated with hepatotoxicity, including dianthrones X7, X8, X9, X12, X13, X14, X15, and X16; anthraquinone glycosides X3, X4, and X6; stilbene glycosides X10 and X11; and flavanols X1, X2, and X5.

It was noteworthy that the dianthrones were the first compounds found by our team to have hepatotoxicity (Yang et al., 2021). The cis- and trans-structures of X7 were shown to have IC_50_ values of 10.98 and 15.45 µM, respectively, in the HepaRG cytotoxicity evaluation. The 96-h LD_50_ of (*cis/trans*) X7 in zebrafish embryos was 1.79 and 1.70 µM (Yang J. B. et al., 2018). X7 exhibited hepatotoxicity at a relatively low concentration of 0.5 mg/L in a zebrafish hepatotoxicity model (Li et al., 2020). X12 exhibited weak hepatotoxicity in L02 cells using the CCK-8 assay (Yang et al., 2016). Moreover, the 96-h LD_50_ of X12 (C4) was 3.39 µM in zebrafish embryos, and a delayed appearance of liver yolk sacs in zebrafish occurred at 0.25 mg/L, indicating definite hepatotoxicity (Yang J. B. et al., 2018). X13 manifested moderate cytotoxicity with IC_50_ values of 29.7–31.1 µM against KB tumor cell lines (Yang J. et al., 2018). The hepatotoxicity of other dianthrones still needs further investigation.

Regarding the screened anthraquinone glycoside components, studies have shown that X3 displayed moderate hepatotoxicity with an IC_50_ value of 71.62 µM in HepG2 cells (Hanh et al., 2021). The 96-h LD_50_ of X3 in zebrafish embryos was 1.10 µM (Yang J. B. et al., 2018). In addition, X3 exhibited zebrafish hepatotoxicity at a low concentration of 0.25 mg/L (Li et al., 2020). However, the structure of X4 is similar to that of X3, with the methoxy group changed to the hydroxyl group on the benzene ring. Moreover, X6 was demonstrated to have strong embryotoxicity and hepatotoxicity in zebrafish in the toxicity test (Yang J. B. et al., 2018; Li et al., 2020). In addition, X6 inhibited the mRNA expression of CYP1A2 and CYP2C and moderately inhibited the activity of UDP-glucuronosyl transferase (UGT1A1), which was suspected to contribute to hepatotoxicity (Jiang et al., 2022).

The hepatotoxic components of the stilbene glycosides screened were 2,3,5,4′-tetrahydroxystilbene-2-*O-β*-D-(2-*O*-monogalloylesters)-glucopyranoside (X10) and polygonibene E (X11). X10 is a stilbene glycoside, and X11 is a stilbene glycoside dimer. At present, few pharmacological studies have been conducted on the above two stilbene glycoside components. However, some studies have reported that the stilbene glycoside component 2,3,5,4′-tetrahydroxystilbene-2-*O-β*-D-glucoyranoside could be a risk factor for hepatotoxicity in PM, which indicates that there may be some potential for hepatotoxicity of stilbene glycosides (Meng et al., 2017).

Regarding the flavanol compounds X1, X2, and X5, oxidation and polymerization have been reported to be the main reasons for the reduction of catechins and flavonoids after processing (Xiang et al., 2021). It has been stated that these polyphenols cause different forms of toxicity, including organ toxicity, genotoxicity, mutagenicity, and cytotoxicity (Islam et al., 2021). For instance, studies have shown that catechin (X1) has antitumor effects and can induce tumor cell apoptosis on account of certain cytotoxicity (Miyamoto et al., 2004). In addition, studies have reported that epicatechin has a concentration-dependent inhibitory effect on tumor cell proliferation and promotes cell death through apoptosis (Varela-Castillo et al., 2018). Epicatechin-3-O-gallate (ECG, X5) induced apoptosis through a TGF-beta superfamily protein, NAG-1 (nonsteroidal antiinflammatory drug-activated gene) (Baek et al., 2004). ECG is a strong inducer of NAG-1, and action on HCT-116 cells leads to an increase in the G (1) phase, leading to cleavage of polyribose polymerase, a phenomenon consistent with apoptosis. In addition, ECG has also been shown to be cytotoxic and hepatotoxic *in vivo* and highly toxic to HSC-2 cancer cells (Babich et al., 2005; Galati et al., 2006).

Other studies have shown that emodin, chrysophanol, and physcion anthraquinones in PM could affect bile acid homeostasis and cause hepatotoxicity (Kang et al., 2022). Some studies also concluded that *cis*-2,3,5,4′-tetrahydroxy-trans-stilbene-2-*O-β*-D-glucoside (*cis*-TSG) in PM led to hepatotoxicity through mitochondrial injury (Liu et al., 2022). In addition, cis-TSG was shown to be more closely related to immunological idiosyncratic hepatotoxicity (Meng et al., 2017). Other views also suggested that the synergy between stilbenes and emodin derivatives contributed to hepatotoxicity of PM (Zhang et al., 2020).

In summary, the 16 chemical components all had different degrees of hepatotoxicity and may be responsible for the hepatotoxicity of PM through a synergistic effect. Among these compounds, the three more typical compounds—emodin dianthrones, emodin-8-*O-β*-D-glucopyranoside, and PM 14–17—showed strong hepatotoxicity in different models. They may be the key hepatotoxic components in PM. However, there were still many limitations in our experiments, such as the toxicity evaluation involving only *in vitro* cells. In addition, the screened hepatotoxic compounds lacked standards, and no further toxicity validation was performed.

## 5 Conclusion

The complexity and diversity of Chinese medicinal components make the discovery of toxic components in Chinese medicine a challenging task. This study integrated a progressive strategy to explore the hepatotoxic components in PM. First, 112 constituents of PM were characterized using UPLC-Q-TOF-MS. Second, plant metabolomics was used to screen for differential components between raw and processed PM. Third, the pseudotargeted mass spectra of the 16 components of 50 batches of PM were established. Then, the hepatotoxicity of 50 batches of PM was evaluated in two hepatocytes. At last, based on three models, GRA, OPLS, and BP-ANN, a spectrum–effect relationship was established to determine the hepatotoxic components in PM. As a result, 16 components with potential hepatotoxicity were found, among which emodin dianthrones, emodin-8-*O-β*-D-glucopyranoside, and PM 14-17 were more significantly prominent. These three markers could be used as hepatotoxic markers in PM as well as for in-depth pharmacological and toxicological studies.

## Data Availability

The original contributions presented in the study are included in the article/Supplementary Material; further inquiries can be directed to the corresponding authors.
