# Supplementary material for: A stepwise strategy integrating metabolomics and pseudotargeted spectrum–effect relationship to elucidate the potential hepatotoxic components in Polygonum multiflorum
Source: Front Pharmacol. 2022 Aug 26;13:935336. doi: 10.3389/fphar.2022.935336 (PMC9459084; doi:10.3389/fphar.2022.935336)
Supplement: Supplementary file 1 [file DataSheet1.docx]

Supplementary Material

Table S1. The detailed information of Sample A

| Samples | Location | Samples | Location |
| --- | --- | --- | --- |
| RPM-1 | Bozhou, Anhui, China | PPM-1 | Changsha, Hunan, China |
| RPM-2 | Dingxi, Gansu, China | PPM-2 | Bozhou, Anhui, China |
| RPM-3 | Beijing, China | PPM-3 | Lijiang, Guangxi, China |
| RPM-4 | Bozhou, Anhui, China | PPM-4 | Anguo, Heibei, China |
| RPM-5 | Xianyang, Shanxi, China | PPM-5 | Zhangshu, Jiangxi, China |
| RPM-6 | Bozhou, Anhui, China | PPM-6 | Chengdu, Sichuan, China |
| RPM-7 | Taizhou, Jiangsu, China | PPM-7 | Guigang, Guangxi, China |
| RPM-8 | Tianshui, Gansu, China | PPM-8 | Shiyan, Hubei, China |
| RPM-9 | Bozhou, Anhui, China | PPM-9 | Zhangshu, Jiangxi, China |
| RPM-10 | Bozhou, Anhui, China | PPM-10 | Kunming, Yunnan, China |
| RPM-11 | Zunyi, Guizhou, China | PPM-11 | Anguo, Hebei, China |
| RPM-12 | Sichuan, China | PPM-12 | Luoyang, Henan, China |
| RPM-13 | Shijiazhuang, Hebei, China | PPM-13 | Kunming, Yunnan, China |
| RPM-14 | Longde, Ningxia, China | PPM-14 | Chengduo, Sichuan, China |
| RPM-15 | Puyang, Henan, China | PPM-15 | Bozhou, Anhui, China |
| RPM-16 | Yuncheng, Shanxi, China | PPM-16 | Bozhou, Anhui, China |
| RPM-17 | Chengdu, Sichuan, China | PPM-17 | Nantong, Jiangsu, China |
| RPM-18 | Shanghai, China | PPM-18 | Shanghai, China |

Table S2. The detailed information of Sample B

| Samples | Location | Samples | Location |
| --- | --- | --- | --- |
| S-1 | Congjiang, Guizhou, China | S-26 | Henan, China |
| S-2 | Xingge, Anhui, China | S-27 | Sichuan, China |
| S-3 | Bozhou, Anhui, China | S-28 | Sichuan, China |
| S-4 | Xinjiang, China | S-29 | Bozhou, Anhui, China |
| S-5 | Yunnan, China | S-30 | Guangdong, China |
| S-6 | Guangxi, China | Z-1 | Bozhou, Anhui, China |
| S-7 | Deqing, Guangdong, China | Z-2 | Bozhou, Anhui, China |
| S-8 | Bozhou, Anhui, China | Z-3 | Bozhou, Anhui, China |
| S-9 | Bozhou, Anhui, China | Z-4 | Bozhou, Anhui, China |
| S-10 | Bozhou, Anhui, China | Z-5 | Lijiang, Yunnan, China |
| S-11 | Bozhou, Anhui, China | Z-6 | Sichuan, China |
| S-12 | Guangxi, China | Z-7 | Henan, China |
| S-13 | Sichuan, China | Z-8 | Henan, China |
| S-14 | Lijiang, Yunnan, China | Z-9 | Henan, China |
| S-15 | Bozhou, Anhui, China | Z-10 | Guizhou, China |
| S-16 | Guangdong, China | Z-11 | Xinjiang, China |
| S-17 | Sichuan, China | Z-12 | Bozhou, Anhui, China |
| S-18 | Guizhou, China | Z-13 | Yunnan, China |
| S-19 | Sichuan, China | Z-14 | Bozhou, Anhui, China |
| S-20 | Henan, China | Z-15 | Xinjiang, China |
| S-21 | Bozhou, Anhui, China | Z-16 | Yunnan, China |
| S-22 | Guangdong, China | Z-17 | Henan, China |
| S-23 | Hunan, China | Z-18 | Henan, China |
| S-24 | Guangdong, China | Z-19 | Sichuan, China |
| S-25 | Guangdong, China | Z-20 | Sichuan, China |

Table S3. IC50 values of 50 batches of raw/processed PM on HepG2 and L02 cells

| Samples | IC_50_(µg/mL) | | Samples | IC_50_(µg/mL) | |
| --- | --- | --- | --- | --- | --- |
|  | HepG2 | L02 |  | HepG2 | L02 |
| S-1 | 180 | 249 | S-26 | 146 | 113 |
| S-2 | 596 | 207 | S-27 | 67 | 86 |
| S-3 | 178 | 238 | S-28 | 71 | 94 |
| S-4 | 228 | 350 | S-29 | 526 | 346 |
| S-5 | 100 | 144 | S-30 | 94 | 68 |
| S-6 | 510 | 493 | Z-1 | 1045 | 655 |
| S-7 | 146 | 176 | Z-2 | 1316 | 569 |
| S-8 | 226 | 341 | Z-3 | 1265 | 559 |
| S-9 | 374 | 225 | Z-4 | 1834 | 668 |
| S-10 | 641 | 186 | Z-5 | 1012 | 866 |
| S-11 | 154 | 126 | Z-6 | 806 | 555 |
| S-12 | 219 | 345 | Z-7 | 970 | 679 |
| S-13 | 76 | 175 | Z-8 | - | 962 |
| S-14 | 71 | 84 | Z-9 | 722 | 584 |
| S-15 | 161 | 186 | Z-10 | 1177 | 940 |
| S-16 | 202 | 275 | Z-11 | 1526 | 604 |
| S-17 | 169 | 133 | Z-12 | 1909 | 559 |
| S-18 | 300 | 456 | Z-13 | 935 | 582 |
| S-19 | 661 | 643 | Z-14 | 1399 | 756 |
| S-20 | 60 | 143 | Z-15 | 1340 | 1513 |
| S-21 | 163 | 134 | Z-16 | 1141 | 1040 |
| S-22 | 853 | 716 | Z-17 | 1539 | 1010 |
| S-23 | 81 | 120 | Z-18 | 1024 | 795 |
| S-24 | 113 | 185 | Z-19 | 628 | 376 |
| S-25 | 1071 | 464 | Z-20 | 936 | 425 |


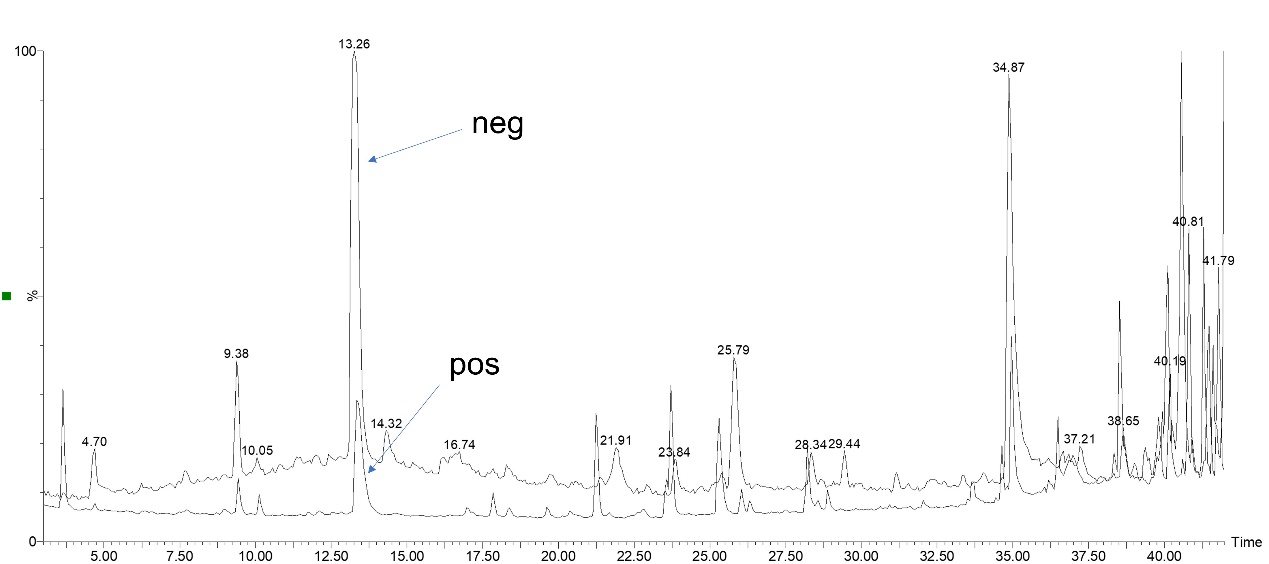


Figure S1. the chromatogram of neg- PM (upper) and pos PM (down)


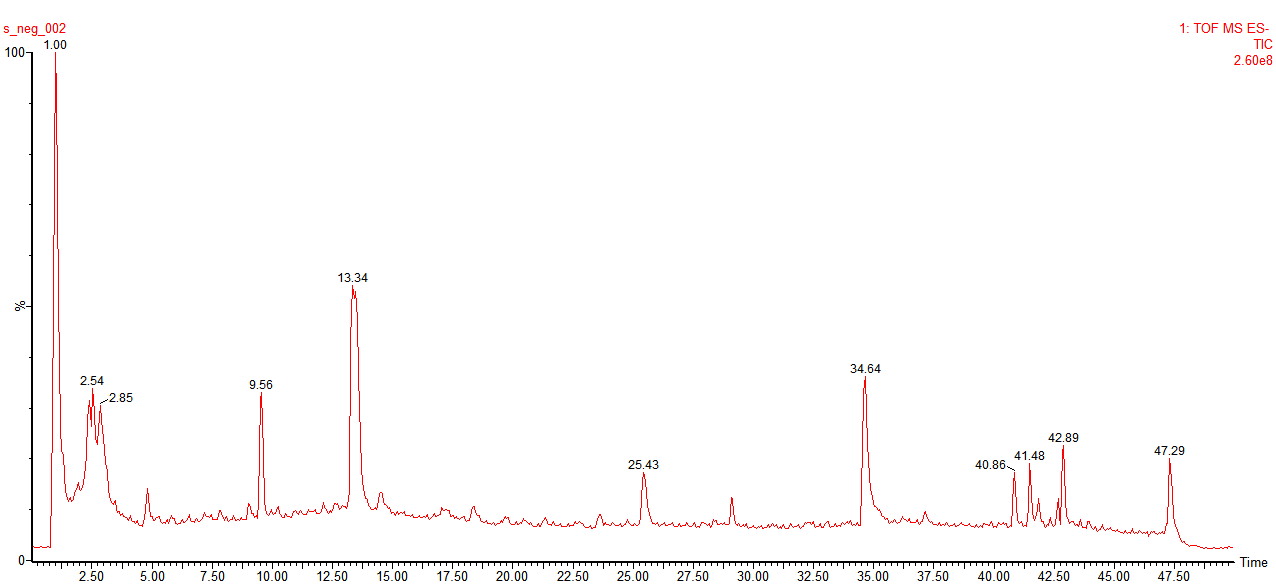

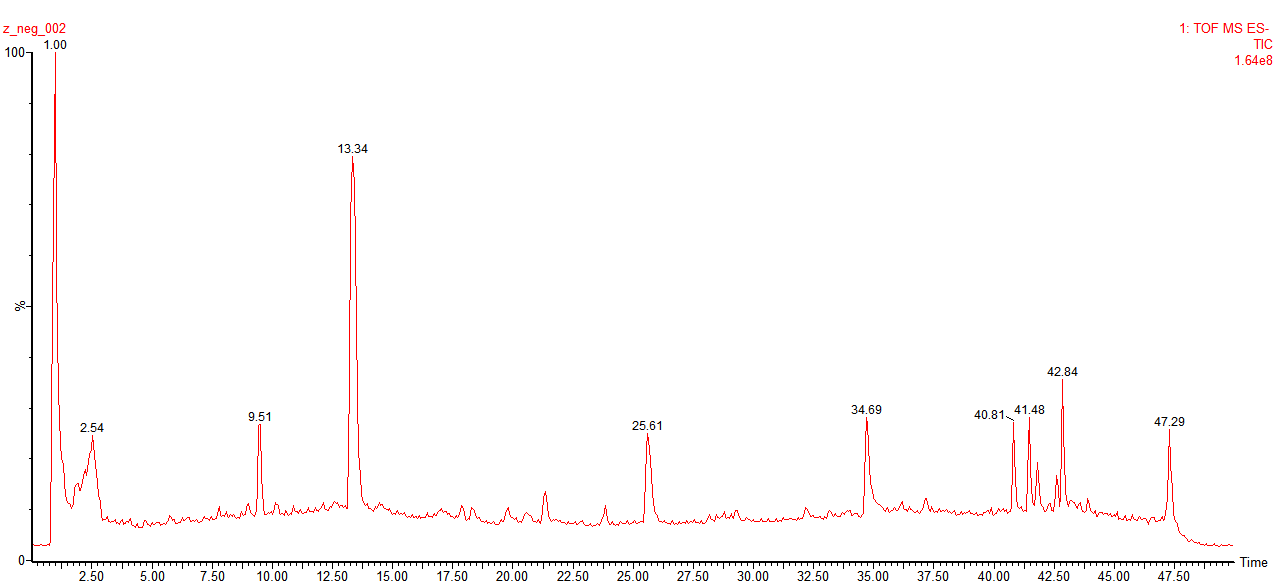


**A**

**B**

Figure S2. the chromatogram of raw PM (A) and processed PM (B)

**
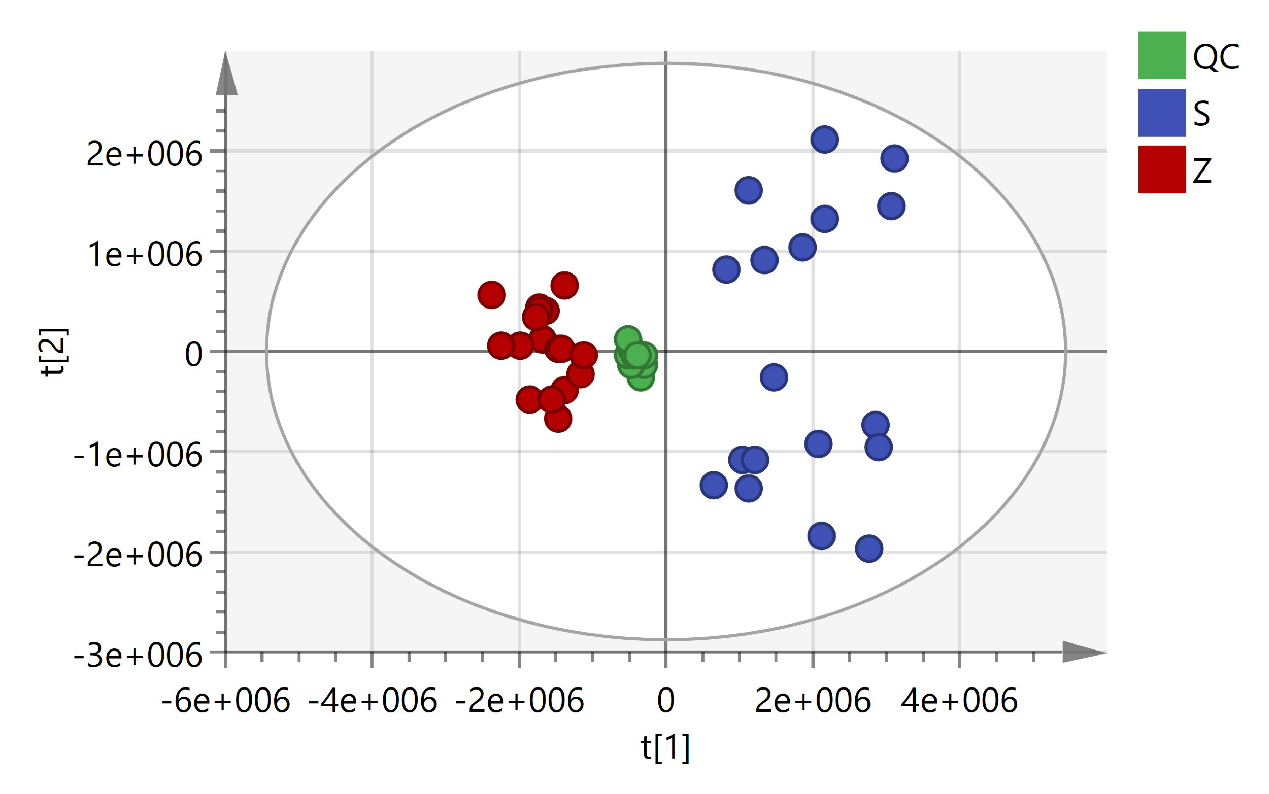
**

Figure S3. PCA score plot of PM samples (QC: quality control; S: raw PM; Z: processed PM)


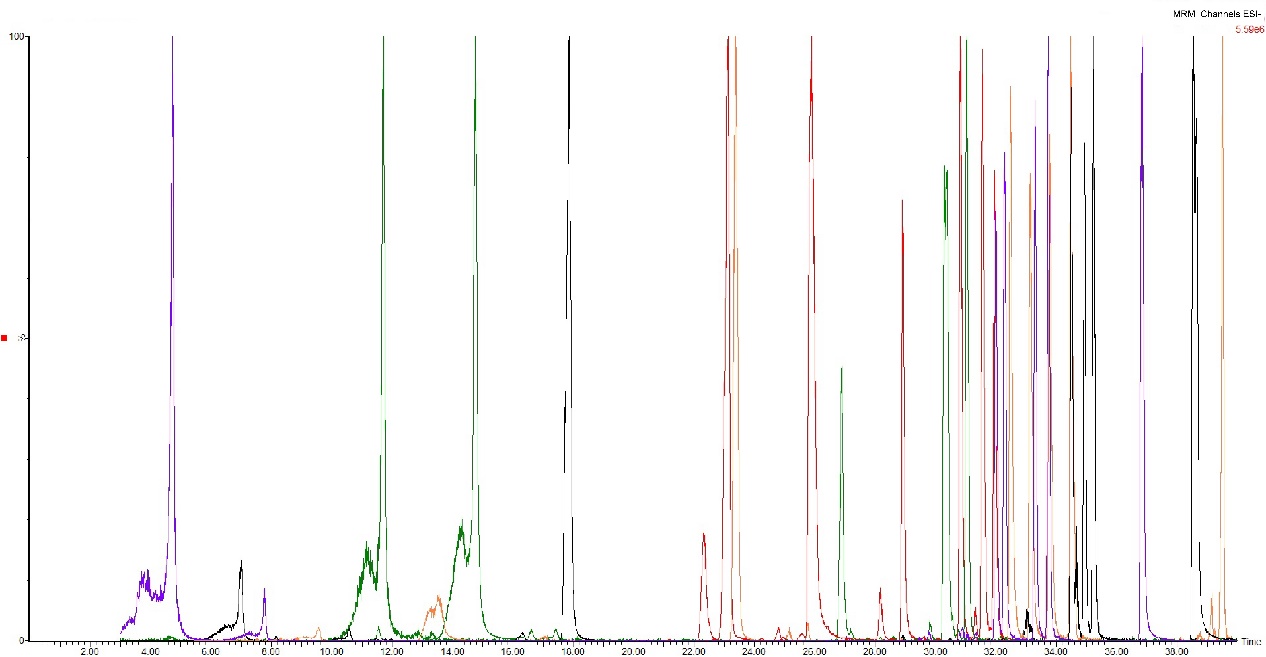


Figure S4. The MRM spectra of 16 target compounds
